# Perspectives of skilled birth attendants and pregnant women regarding episiotomy: a quantitative approach

**DOI:** 10.4314/ahs.v21i3.47

**Published:** 2021-09

**Authors:** Dokuba Tex-Jack, Chinemerem Eleke

**Affiliations:** 1 University of Port Harcourt, African Centre of Excellence, Centre for Public Health and Toxicological Research; 2 University of Port Harcourt, Department of Nursing

**Keywords:** Episiotomy, birth, pregnant women, vagina, Nigeria

## Abstract

**Background:**

The World Health Organization recommended less than 10% episiotomy rate for Skilled Birth Attendants (SBAs) and hospitals in 1996. More than two decades afterwards, some health facilities are still grappling with meeting the set target.

**Objectives:**

This study assessed the perspectives of SBAs and pregnant women regarding episiotomy in a Nigerian university teaching hospital.

**Methods:**

A cross-sectional design was employed. Census sampling was used to select 19 SBAS and 973 vaginal birth records from 2019, while consecutive sampling technique was used to enrol 134 consenting pregnant women obtaining antenatal services in the facility. Data was collected using a three part instrument involving a data extraction sheet, episiotomy practice questionnaire for SBAs, and feelings about episiotomy questionnaire for pregnant women. Assembled data were summarised with descriptive statistics.

**Results:**

The episiotomy rate was 345(35.5%). About 266 (77.1%) of first time mothers (primips) and 79(22.9%) of nonfirst time mothers (multips) received episiotomy. Ten (52.6%) of the SBAs were unsure of any evidence supporting routine episiotomy. All the 19(100%) SBAs reported that there was no existing facility-based policy regarding routine episiotomy. Seventy five (56%) of the pregnant women reported feeling generally bad about episiotomy. One hundred and one (82.3%) of them hinted that they will not feel satisfied if they were given episiotomy with the reason that it ensures quick vaginal birth.

**Conclusion:**

The rate of episiotomy was higher than global recommended standards and primips are disproportionately affected. If organised by professional societies, more scientific conferences on limiting episiotomy might remedy this situation.

## Introduction

Episiotomy has been in use by midwives and obstetricians since the twentieth century. It is a cut into the perineum by obstetrical caregivers that was historically thought to expedite vaginal birth^1^,^2^. Over the years, mediolateral episiotomy became widespread and was at some point used on routine basis by midwives and obstetricians^3^. In recent times however, rigorous empirical research have queried the clinical benefits of routine episiotomy^4^,^5^. Many randomize control trials concluded that episiotomy revealed no significant benefit to suggest its routine use by obstetric caregivers on women undergoing virginal birth^6^. Some studies unravelled that episiotomy increased the chances of perineal and sphincter damage^7^. Based on this, several researchers suggested restrictive episiotomy over routine episiotomy^8^,^9^. More so, other researchers suggested that episiotomy should be avoided where possible^10^. Consequently, the World Health Organization (WHO) shifted from its 10% episiotomy rate recommendation of 1996, to a no practice of episiotomy policy^11^. Although a uniform recommended episiotomy rate across professional bodies within the five continents have not been achieved, most organisations accept a less than 10% episiotomy rate as ideal^1^,^4^. Despite global set standards, commensurately low episiotomy rates of episiotomy have not been observed is some continents especially Africa and East Asia^12^,^13^.

Since the beginning of the twenty first century, the practice of routine episiotomy by midwives and obstetricians have remained under public scrutiny^14^. The alignment of the clinicians reasons for episiotomy with maternal preference for episiotomy have generated discourse among researchers and clinicians globally^15^. Consequently, a study of this nature which is aimed at examining the perspectives of skilled birth attendants and pregnant women regarding episiotomy in health facilities within Africa is justified.

In Nigeria, the Nigerian Association of Obstetricians and Gynaecologists accepts a 10% episiotomy rate as adequate^16^,^17^. Prior to 2019, documented episiotomy rates in several university teaching hospitals in different parts of Nigeria ranged between 34% to 41%^16^,^17^. This fore mentioned calls for concern, hence the research team in this study were motivated to investigate the perspectives of skilled birth attendants and pregnant women regarding episiotomy in a university teaching hospital in south-southern Nigeria.

## Methods

### Design and participants

This cross-sectional study was carried out in 2020, on 973 vaginal birth records, 19 skilled birth attendants (SBAs) and 134 pregnant women attending antenatal clinic in one particular university teaching hospital located in south-southern Nigeria. Census sampling was used to select all available records of spontaneous vaginal birth of singletons between January and December of 2019 and all 19 SBAs practicing in the labour ward of the hospital, while 134 consecutively accessible and consenting pregnant women were selected. The sample size for the pregnant women was determined using the Fisher (1998) formula for sample size calculation for cross-sectional studies:; where, n = minimum sample size, Z = constant at 95% confidence interval (1.96), P = proportion of best guess (50%), and d = precision (0.05). A minimum sample size of 384 was computed. For the reason that the population of registered pregnant women in the antenatal clinic of the hospital was 205 (i.e. less than 10,000) the alternative Fisher (1998) formula for sample size reduction was applied:; where nf = Final sample size, n = minimum sample size (384), and N = the population size (205). A final sample size of 134 for clients was computed.

### Instruments

A three part instrument was utilised for data collection. Part one was a data extraction sheet which tapped information on frequency of episiotomies performed in the year 2019. Part two was a structured questionnaire for SBAs, and it assessed issues surrounding their episiotomy practice. It was a 13-item questionnaire with two sections (A and B). Section A had five items which elicited the socio-demographic profile of the SBAs. Section B had eight items that assessed their practice of episiotomy. Part three was a structured questionnaire for pregnant antenatal women and it examined their feelings regarding episiotomy. It was an 8-item questionnaire with two sections (C and D). Section C had three items that drew out information on socio-demography of the pregnant women. Section D had five items that assessed their feelings about episiotomy practice.

### Data collection

Data collection started after permission had been obtained from the administration of the hospital. All data were assembled between January and June 2020. Vaginal birth records from 2019 were examined and relevant data were extracted using part one of the study instrument. The SBAs were approached during break hours. The purpose of the study was explained to them and they were given part two of the study instrument to respond in their chosen private setting and return it to the research team within 30 minutes. The pregnant antenatal women were approached at the antenatal care unit of the hospital. The purpose of the study was explained to them and part three of the study instrument was given to them to respond and return it to the research team within 30 minutes.

### Ethical considerations

The protocol for this study was reviewed and approved by the University of Port Harcourt Research Ethics Committee (Protocol ID: G2018/PUT/MAS/MMW/FT/034). Administrative permission was obtained before obtaining required data. The purpose of the study was explained to all participants as well as the voluntary nature of this study. Informed written consent was obtained from participants who were willing to participate in the study. The participants were allowed to fill their questionnaire in their chosen location for privacy. The participants were assured that any information given was for academic purpose and not to indict them. Discussion with hospital authorities came to an agreement that the name of the hospital of study will not be put in print. All obtained data were protected and considered confidential.

### Data analyses

Data entry and analyses was done with the aid of Statistical Package for Social Sciences version 21 (SPSS Inc. Chicago, IL, USA). All collected and collated data were presented using descriptive statistics (mean, standard deviation, frequency and percentage).

## Results

Table 1 summarised the socio-demographic characteristics of the participants (SBAs and pregnant women), and it showed that the skilled birth attendants had a mean age of 36.8(6.4) years. Nine (47.4%) of them were aged between 36 and 44 years, and 13(68.4%) were females. Nine (47.4%) of them were midwives with diploma level education and a mean of 7.0(2.9) years clinical practice experience. The mean years of experience on their current job in the labour ward was 5.3(2.4) years. Additionally, all the antenatal women were married, and had a mean age of 31.2(5.9) years. Eighty one (60.4%) of them were aged between 21 and 32 years and 92(68.7%) had experienced episiotomy in previous vaginal births.

**Table 1:** Socio-demographic characteristics of participants

Variable	f	%	Mean(SD)
**Skill Birth Attendants N = 19**			
**Age**			
18–26 years	1	5.3	
27–35 years	7	36.8	
36–44 years	9	47.4	
45–53 years	2	10.5	
Mean			36.8(6.4)
**Gender**			
Male	6	31.6	
Female	13	68.4	
**Highest educational qualification**			
Nursing Diploma (RM)	9	47.4	
Nursing Bachelors (BSN, RM)	2	10.5	
Nursing Masters (MSN, RM)	1	5.2	
Medicine Bachelors (MBBS)	7	36.8	
**Years of clinical experience**			
1–5 years	7	36.8	
5–10 years	9	47.4	
10–15 years	3	15.8	
Mean			7.0(2.9)
**Years of labor ward experience in RUSTH**			
1–5 years	13	68.4	
5–10 years	5	26.3	
10–15 years	1	5.3	
Mean			5.3(2.4)
**Pregnant antenatal women n = 134**			
**Age**			
21–32 years	81	60.4	
33–44 years	53	39.6	
Mean			31.2(5.9)
**Marital status**			
Married	134	100	
**Previous experience with episiotomy**			
Previously had episiotomy	92	68.7	
Have never had episiotomy	42	31.4	

Table 2 summarised the episiotomy rate in 2019, and showed that it was high at 35.5%. About 266(77.1%) of first time mothers (primips) and 79(22.9%) of non-first time mothers (multips) received episiotomy in that year. Table 3 summarised the practice of episiotomy among the SBAs, and it revealed that 9(47.3%) of them reported to practice episiotomy based on indication. Six (31.6%) reported that they assess degree of perineal stretch before carrying out any episiotomy procedure. Ten (52.6%) of the respondents were not sure if empirical evidence supports episiotomy practice, meanwhile they reported that there was no existing institutional policy on episiotomy in the facility. More than half of them reported that tight perineum (17; 89.5%), shoulder dystocia (15; 78.9%), instrumental delivery (12; 63.1%) and breech presentation (11; 57.9%) were the major indications for their performance of episiotomy. Most of them reported that their reason for episiotomy practice was to prevent perineal tear (19; 100%) and prolonged second stage of labour (12; 63.1%). Eighteen (94.7%) reported that they involve pregnant women in decisions of episiotomy by obtaining informed consent prior to episiotomy. Furthermore, sixteen (84.2%) reported that they give local anaesthesia before carrying out episiotomy.

**Table 2:** Episiotomy rate in 2019

	N = 973					

Year	Total Vaginal Births	Total Episiotomies	Episiotomy rate	Episiotomy on Primips	Episiotomy on Multips	Interpretation
	f	f	%	f(%)	f(%)	
**2019**	973	345	35.5	266(77.1)	79(22.9)	High

**Decision rule**: Episiotomy rate ≤ 10% = Low rate of episiotomy, > 10% = High rate of episiotomy

**Table 3:** Practice of Episiotomy among Skilled Birth Attendants N = 19

No.	Interview Items	f	%
**1.**	**What kind of episiotomy practice do you employ in your facility?**		
	a. Restrictive episiotomy	1	5.3
	b. Routine episiotomy	3	15.8
	c. Selective episiotomy	6	31.6
	d. Episiotomy based on Indication	9	47.3
**2.**	**What assessment do you do before episiotomy?**		
	a. Proportion of fetal head to pelvis test	2	10.5
	b. Assessment of crowning	4	21.1
	c. Degree of perineum stretch	6	31.6
	d. Size of the baby	4	21.1
	e. Prematurity	2	10.5
	f. Breech/shoulder dystocia	1	5.3
**3.**	**Is there research evidence to support episiotomy practice?**		
	a. Yes	6	31.6
	b. Not sure	10	52.6
	c. No	3	15.8
**4.**	**Is there any institutional policy on episiotomy in your facility?**		
	a. No	19	100
**5.**	**What indications mostly necessitate your practice episiotomy in** **this labor ward? Multiple choice required**		
	a. Tight perineum	17	89.5
	b. Primigravida	8	42.1
	c. Shoulder dystocia	15	78.9
	d. Breech presentation	11	57.9
	e. Instrumental delivery	12	63.1
	f. Patient's choice	2	10.5
	g. Female genital cutting/mutilation	-	-
**6.**	**What are your most important rationales for performing episiotomy** **procedure? Multiple choice required**		
	a. Protects against perineal tear	19	100
	b. Prevents prolonged second stage of labor	12	63.1
	c. Prevents fetal distress	7	36.8
	d. Results in better perineal healing	4	21.0
	e. Prevents pelvic floor dysfunction	7	36.8
	f. Prevents urinary inconsistencies	1	5.3
	g. Prevents fetal inconsistencies	1	5.3
	**Do you obtain consent from patient before episiotomy?**		
	a. No	1	5.3
	b. Yes	18	94.7
	**Do you give local anaesthesia before episiotomy?**		
	**a. No**	3	15.8
	**b. Yes**	16	84.2

Table 4 summarized pregnant women's feelings about episiotomy, where more than half of the antenatal women (75; 56%) reported that they feel generally bad towards episiotomy. Thirty six (27%) of them responded that women should not have episiotomy during vaginal birth. Most of them (98; 73.1%) were of the feeling that a pregnant woman should be pre-informed before an episiotomy is done. Ninety one (67.9%) had the feeling that the choice to give episiotomy should not reside mainly with the midwife and/or doctor. Most of the clients (111; 82.3%) hinted that they will not feel satisfied if they were given episiotomy with the reason that it ensures quick vaginal birth.

**Table 4:** Feelings about episiotomy among pregnant women n = 134

No.	Interview Items	f	%
**1.**	**How do you generally feel about episiotomy**?		
	a. Bad	75	56.0
	b. Undecided	17	46.7
	c. Good	42	31.3
**2.**	**Who do you feel requires episiotomy?**		
	a. First time mothers	35	26.1
	b. Women with tight vagina	22	14.4
	c. Women having prolonged labor	10	13.4
	d. Women with risk of harm to the fetus	31	23.1
	e. None	36	27.0
**3.**	**Do you feel that a pregnant woman should be pre-informed before an episiotomy?**		
	a. No	36	26.9
	b. Yes	98	73.1
**4.**	**Do you feel that the choice to give episiotomy should reside mainly with the midwife** **and/or doctor?**		
	a. No	91	67.9
	b. Yes	43	32.1
**5.**	**Will you feel satisfied if you were given episiotomy with the reason that it ensures** **quick vaginal birth?**		
	a. No	111	82.3
	b. Yes	23	17.7

## Discussion

This study found that the episiotomy rate of 35.5% in 2019 was higher than the World Health Organization's 2018 recommendation.^11^ This finding agrees with a study set in university teaching hospital in Port Harcourt, south-southern Nigeria which found an episiotomy rate of 22.1%^16^. The similarity in findings could be linked to region where the studies were done. Both studies were conducted in south-southern Nigeria. This may suggest that high episiotomy rates results from the conventional practice among obstetrical caregivers in the region. Nonetheless, this finding was lower than the episiotomy rate of 73.3% noted in a recent Lebanese study^18^. The dissimilarity in findings could be connected to differences in sample size. This study examined a 973 vaginal records from one single year, whereas the Lebanese study assessed 1756 records through six years. A larger sample size perharps offers a more valid conclusion. This finding corobrate a multi-national African study which affirmed that average episiotomy rate in sub-saharan Africa was approximately 25.4%^9^. This finding would imply an urgent need for continued training and workshops for skilled birth attendant on reducing episiotomy practice.

This study noted that the primips were disproportionately given episiotomy by obstetrical caregivers (77%). This finding corroborate a previous Nigerian study which noted that up to 79.4% of women who receive episiotomy are primips^17^. The proximity in results could be linked to the fact that both studies utilised single-centre facility based records. This result also agrees with an Ethiopian study which found that primips were 15 times more likely to get episiotomy compared to multips^13^. This result may suggest a need for stake-holders to enlighten primips on their right to refuse episiotomy where not indicated.

This study revealed that majority of the SBAs reported practicing episiotomy based on indication. This finding did not align with examined vaginal birth records as no clearly stated indication for episiotomy were identified by this research team. Nonetheless, the SBAs reported tight perineum, shoulder dystocia, instrumental delivery and breech presentation to be the major indications for their performance of episiotomy. This finding aligned with a study in Oman, which found that tight perineal tissue and shoulder distocia were mention as indications for episiotomy by midwives and obstetricians^19^. Since the WHO recomends avoidance of episiotomy as no known indication for episiotomy have been empirically demonstrated, the responses of the SBAs was considered inappropriate hence buttressing a need for continuing education and re-training of SBAs. Additionally, this study found that majority of the SBAs were unsure if empirical evidence supports episiotomy practice and had no available Institutional guideline regarding episiotomy practice in the facility. This finding corroborates a Jordanian study which hinted that midwives and obstetricians had little access to training and evidence-based institutional guideline on episiotomy^10^. This finding would suggest that episiotomy is relegated to the discretion and expertise of the caregiver. This finding perharps imply a need for hospitals to develop institution based guidelines geared at checkmating the practice of episiotomy. This study further fond that the SBAs reported that prevention of perineal tear was the main rationale for the practice of episiotomy. Nonetheless, the set of reasons offered by the SBAs were not evidenced by a Canadian study which found that episiotomy increased the risk of obstetric anal sphincter tear by up to 106%^6^.

This study found that more than half of pregnant women feel generally bad towards episiotomy and feel no woman should be given episiotomy. This finding aligned with a Nigerian study which noted that about 56% of pregnant women will advise their relatives against episiotomy. The similarity in findings between the studies was not supprising as data were collected from antenatal women in both studies. Additionally, this study revealed that most pregnant women would not generally feel satisfied if they were given episiotomy with the reason that it ensures quick vaginal birth. This finding would imply that pregnant women would not prefer episiotomy given the chance. This however was in contrast with another Nigerian study which hinted that most (89.9%) pregnant women were willing to give birth in a secondary health facility whether or not they will be given episiotomy. Furthermore, this study found that majority of pregnant women feel that a pregnant woman should be pre-informed before an episiotomy is carried out during vaginal birth, hence suggesting that the choice to give episiotomy should not reside mainly with the midwife and/or doctor. This would suggest that women may see their right to choice of treatment as violated if they were not pre-informed about episiotomy prior to practice^21^.

The major strength of this study is the ability to extract documented episiotomy procedures, and self report perspectives of SBAS and pregnant women for confirmatory evaluation. On the other hand, one limitation of this study was that this study was conducted in one facility, hence the results may not generalise outside the study population.

## Conclusion

The rate of episiotomy was higher than WHO recommended level. SBAS listed some indications for episiotomy which were not based on current WHO guidelines regarding episiotomy and were also not supported by current literature. Compared to multips, primips disproportionately received episiotomy perhaps on routine basis. Pregnant women have negative feelings about episiotomy and feel episiotomy should never be used on women having spontaneous vaginal birth.
